# Genetic nexus of assisted reproduction technique efficacy in infertile women: insights from a comprehensive retrospective study

**DOI:** 10.1007/s13353-025-00961-9

**Published:** 2025-04-03

**Authors:** Fengying Li, Bingqi Ye, Mengsha Chen, Xiaoling Zhou, Lei Yu, Jie Xiang, Xiaobin Ren, Jun Zhang

**Affiliations:** 1 Department of Clinical Laboratory, Sir Run Run Shaw Hospital, Zhejiang University School of Medicine, 3 East Qingchun Road, Hangzhou, 310016 Zhejiang Province People’s Republic of China; 2 Key Laboratory of Precision Medicine in Diagnosis and Monitoring Research of Zhejiang Province, 3 East Qingchun Road, Hangzhou, 310016 Zhejiang Province People’s Republic of China; 3 Sir Run Run Shaw Hospital, School of Public Health, Zhejiang University School of Medicine, Hangzhou, 310058 Zhejiang Province People’s Republic of China; 4 Zhejiang Provincial Center for Disease Control and Prevention, Hangzhou, 310051 Zhejiang Province People’s Republic of China; 5 Institute of Infectious Disease Control and Prevention, Hangzhou Center for Disease Control and Prevention (Hangzhou Health Supervision Institution), 568 Mingshi Road, Hangzhou, 310021 Zhejiang Province People’s Republic of China

**Keywords:** Methylenetetrahydrofolate reductase, Assisted reproduction techniques, Assisted reproduction techniques efficacy, Infertility

## Abstract

Infertility presents a substantial challenge for women of reproductive age, exerting profound effects on both individual well-being and healthcare systems. Despite its critical role in folate and homocysteine pathways, the influence of methylenetetrahydrofolate reductase (MTHFR) on the success of assisted reproductive techniques (ART) remains insufficiently understood. This knowledge gap impedes the development of personalized therapeutic strategies. Our study seeks to elucidate the relationship between *MTHFR* and ART outcomes, exploring potential mediators to enhance treatment efficacy. A cohort of 311 women with infertility was recruited for our study. Multivariate linear models were utilized to evaluate the relationship between the *MTHFR* 677T allele (a missense mutation resulting in an alanine to valine substitution) and the efficacy of ART, including both treatment outcomes and the number of ART cycles required. Sequential mediation analysis was conducted to elucidate the potential mediators influencing ART efficacy. The *MTHFR* 677T allele carried by infertile women was associated with a 17–51% reduction in ART efficacy (*P* < 0.05). This encompassed poorer overall ART outcomes, such as clinical pregnancy and live birth rates, as well as an increased number of ART cycles. Sequential mediation analysis suggested that anti-Müllerian hormone (AMH) and age may act as mediators modulating the impact of the *MTHFR* 677T allele on ART treatment efficacy. This study has unveiled the intricate connection between *MTHFR* 677T allele and ART treatment efficacy in infertile women, shedding light on the mediating role of AMH and age.

## Introduction

Infertility, characterized as the failure to achieve conception following a minimum of 12 months of consistent and unprotected sexual intercourse (Zegers-Hochschild et al. 2017), impacts 8–12% of women in the reproductive age bracket (Agarwal et al. 2021; O'Neill et al. 2018). It frequently gives rise to significant psychological and social distress, thereby imposing a substantial burden on healthcare systems (Carson and Kallen 2021). In more than 85% of female infertility cases, there are identifiable abnormalities in normal physiological functions or underlying conditions, such as obesity and ovulatory dysfunction (Gelbaya et al. 2014). Opting for the early and appropriate treatment of infertility not only offers the potential for a successful pregnancy but also promotes overall health and well-being (Lu et al. 2023; Huang et al. 2023).

In the modern era, there have been remarkable advancements in the accessibility and utilization of a range of advanced assisted reproduction techniques (ART) (Miller et al. 2019). These techniques encompass well-established options such as conventional in vitro fertilization (IVF), intracytoplasmic sperm injection (ICSI), and embryo transfer (ET). The widespread availability of these ART procedures has brought about a revolution in the field of reproductive medicine, resulting in the birth of over 5 million infants globally through more than 2 million treatment cycles annually (Callahan et al. 1994). It is important to note, however, that the effectiveness of ART can vary significantly across different regions, primarily due to the influence of host genetic factors (Geyter 2019; Viera-Molina and Guerra-Martín 2018). Understanding the intricate interplay between these genetic factors and the efficacy of ART has become a paramount concern, particularly for infertile women. Uncovering the impact of host genetic factors on ART outcomes holds great potential for advancing personalized treatment approaches for infertility.

The methylenetetrahydrofolate reductase (*MTHFR*) gene, a pivotal regulator in folic acid metabolism, hosts polymorphisms that have become one of the most frequently reported genetic variations among women of childbearing age. Nevertheless, the precise role of the 677T allele in the *MTHFR* gene, which results in an alanine-to-valine substitution, in determining ART efficacy remains uncertain. While evidence suggests that the presence of the 677T allele, one of the most common missense mutations of the *MTHFR* gene, is associated with reduced levels of AMH, as measured by enzyme-linked immunosorbent assay (ELISA) (Shahrokhi et al. 2017), our understanding of this relationship remains incomplete. Numerous studies have aimed to illuminate the link between *MTHFR* 677T allele and ART outcomes. For example, a prospective study conducted in Scotland involving 602 participants suggested that individuals with either the heterozygous CT or homozygous TT genotype at *MTHFR* 677 allele, demonstrated a higher likelihood of achieving clinical pregnancy and live births (Haggarty et al. 2006). Similarly, a study conducted in Brazil reported higher ART success rates among women with the heterozygous CT or homozygous TT genotype at *MTHFR* 677 allele (D’Elia et al. 2014). Nevertheless, these studies, while significant, have primarily concentrated on the influence of genetic variations on the ultimate results of ART, neglecting their potential impact on the number of ART cycles. This oversight is noteworthy as the number of ART treatment cycles undertaken serves as a robust indicator of both the healthcare-economic burden and the overall efficacy of the ART procedure. Moreover, these studies have, thus far, failed to unveil the potential mediators through which genetic polymorphisms may exert their effects. By solely scrutinizing the connection between *MTHFR* 677T allele and ART outcomes, they provide a somewhat one-sided perspective, rather than offering a comprehensive understanding of ART efficacy by encompassing genetic polymorphisms in conjunction with other critical phenotypic traits. To thoroughly comprehend the intricate interplay between genetic factors and ART success rates, it is imperative to consider the broader context of phenotypic characteristics and their potential mediating roles.

In this study, we investigated the complex relationship between the *MTHFR* 677T allele and the efficacy of ART. We not solely focus on the outcomes of ART but also address the critical aspect of treatment cycles. Furthermore, we embark on a comparative analysis of different types of ART in the context of various genotype conditions. This thorough examination enables us to identify optimal choices for personalized treatment strategies, ultimately enhancing the prospects of success for individuals grappling with infertility.

## Materials and methods

### Study population

**Fig. 1:**
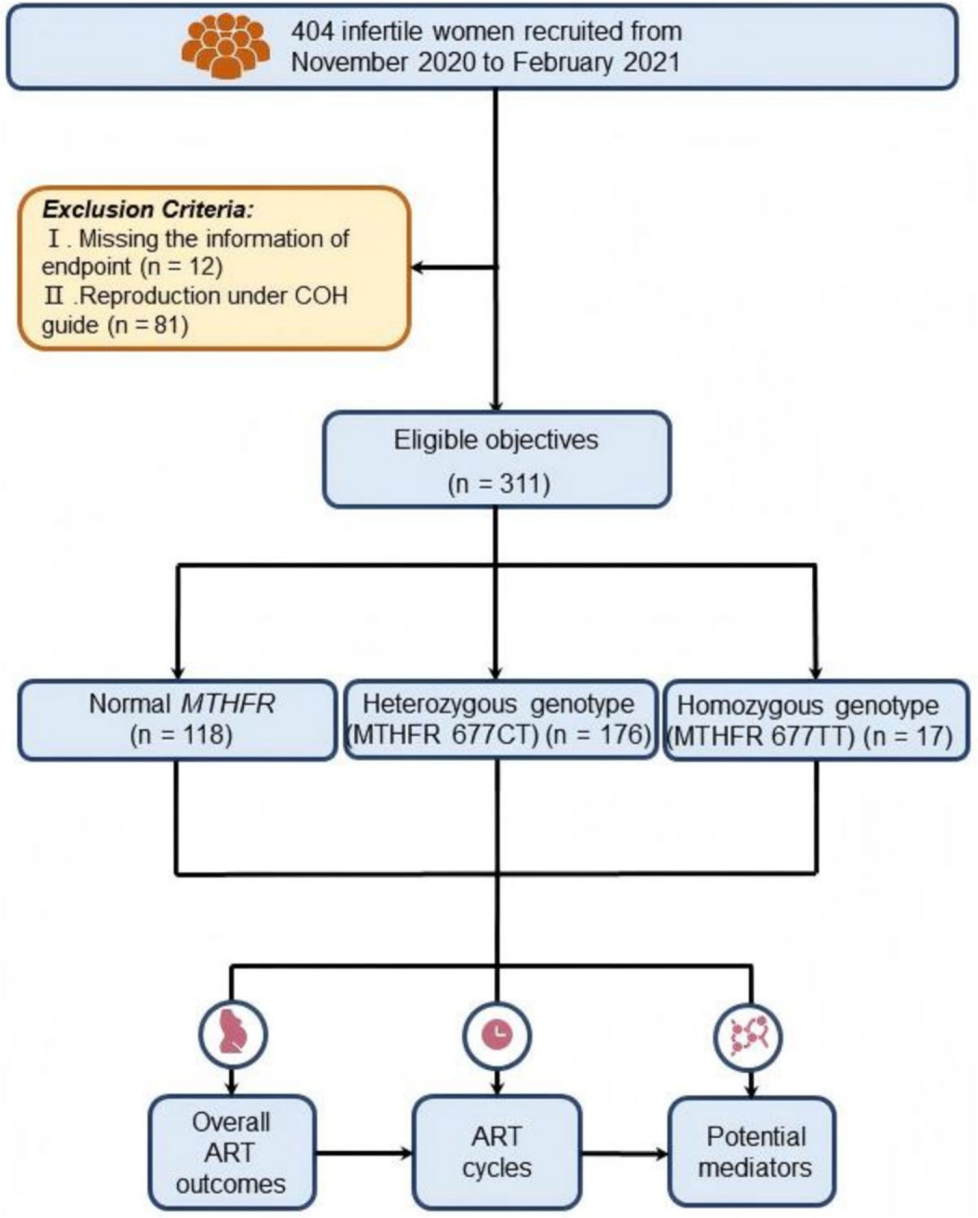
The work diagram of this study

Between November 2020 and February 2021, a total of 404 women seeking infertility treatment were admitted to the Reproductive Medicine Center, Department of Obstetrics and Gynecology, at the Sir Run Run Shaw Hospital of Zhejiang University. Ninety-three participants were excluded from the study due to the following reasons: (1) missing information on the endpoint and (2) a lack of clinical indications for the embryo transfer program, achieving pregnancy under controlled ovarian hyperstimulation (COH) guidance. Based on these exclusion criteria, 311 subjects were selected for observation in consecutive series of ART treatment cycles and subsequent analyses. An overview of the study design is depicted in Fig. 1. Informed consent was obtained from all participants.

### The analysis of the frequency of MTHFR 677 T allele

The frequency of the *MTHFR* 677T allele was assessed in 311 infertile women using pyrosequencing with commercially available genotyping assay kits (Sanji, Changsha, China). Genomic DNA was extracted from peripheral blood following the manufacturer's instructions and served as a template for PCR reactions. Regular PCR was carried out for each DNA sample, involving 35 cycles with the following conditions: 5 min of denaturation at 95 °C, followed by 30 s at 94 °C, 30 s at 60 °C, and 30 s at 72 °C. Before pyrosequencing, the PCR product was biotinylated and attached to Streptavidin-coated Sepharose beads, which were captured using a vacuum tool. Single-stranded DNA was generated through denaturation steps and subsequently released into the pyrosequencing reaction plate. The pyrosequencing reaction was conducted on the PyroMark Q24 instrument following the manufacturer’s instructions. The analysis of genotyping results was performed using PyroMark Q24 2.0.6 software.

### Ovarian stimulation protocol

A personalized controlled ovarian hyperstimulation protocol was implemented for each individual. Essentially, the selection of either a long gonadotrophin-releasing hormone (GnRH) agonist protocol or a short GnRH agonist protocol was based on clinical indications. Ovarian stimulation involved the administration of recombinant FSH (rFSH, Gonal-F; Serono Laboratories, Switzerland) at a daily dosage ranging from 150 to 300 IU, commencing on the third or fifth day of the menstrual cycle. Follicular development was monitored through ultrasound scanning, and when the leading follicle reached a diameter of 16–18 mm, β Human chorionic gonadotropin (β-HCG) (Serono; Denmark; 6500–10,000 IU) was administered to induce ovulation. Transvaginal oocyte retrieval was performed 36 h after the immediate injection of β-HCG. The procedures of IVF/ICSI were carried out in accordance with standard practices. Following oocyte insemination, embryo culture was initiated, followed either by fresh embryo transfer or frozen-thawed embryo transfer, based on clinical conditions. The luteal phase was supported with progesterone in oil at a dosage of 40 mg/day, and treatment continued until fetal heart activity was visualized on ultrasound.

### Evaluation of the ART efficacy

Pregnancy was considered to have occurred when the serum β-HCG level exceeded 10 IU/L 12 days after embryo transfer (Al Mamari et al. 2019). Clinical pregnancy was defined as the confirmation of at least one gestational sac through ultrasound observation 35 days after embryo transfer. Biochemical pregnancy was characterized by an initial examination of the β-HCG level over 10 IU/L and a subsequent measurement (at least 2 days apart) higher than the initial one; however, it did not progress to clinical pregnancy. Abortion was identified as a pregnancy that failed to progress after the detection of an intrauterine gestational sac through pelvic ultrasonography. Live birth referred to the ultimate delivery of at least one neonate. If the fresh embryo transfer is unsuccessful, subsequent attempts will be tailored to the patient’s individual circumstances until a live baby is born or until the conclusion of the designated follow-up period. The efficacy of ART encompasses the number of embryo transfer cycles and the corresponding outcomes of the ART treatment. The outcomes of ART treatment were characterized by improved pregnancy-related indicators, including clinical pregnancy and live birth.

### Assessment of covariates

Venous blood samples were systematically collected from all participants before commencing their initial ART treatment cycle. These blood samples were promptly processed and examined at the Clinical Laboratory of Sir Run Run Shaw Hospital, Zhejiang University. The analysis of β-HCG levels utilized a chemiluminescence immunoassay, employing the UniCel DxI 800 system by Beckman Coulter (Brea, USA). Measurements of urea and creatinine were conducted using a Beckman Coulter AU5800 analyzer (Beckman Coulter, Brea, USA). The detection of Anti-β2 glycoprotein-I (aβ2GPI) and anticardiolipin (aCL) IgG/A/M antibodies was accomplished through an iFLASH3000 immuno-analyzer by YHLO Biotech (Shenzhen, China). D-Dimers (D-Di) were quantified using a latex immunoassay method on the STAGO system by Diagnostical Stago (Asnières, France). Carbohydrate antigen 125 (CA125) and anti-Müllerian hormone (AMH) levels were measured employing an electrochemiluminescence assay, facilitated by Roche’s eCobas e602 fully automated platform (Roche Diagnostics, Rotkreuz, Switzerland).

Age and body mass index (BMI) were obtained from medical records. BMI (kg/m^2^) was categorized into lean (≤ 18.5), normal weight (18.5–24), and overweight or obesity (> 24).

### Statistical analyses

Categorical variables were presented as frequencies and percentages and analyzed using the Chi-squared test. Since all continuous variables were non-normally distributed, they were reported as median (interquartile range) and analyzed using the Wilcoxon test and Mann–Whitney test. For the multivariable logistic regression analysis, we established three models: Model 1 (crude model) without any adjustments; Model 2, which was adjusted for age and BMI; Model 3, which included covariates from Model 2 as well as routine laboratory tests such as urea, creatinine, D-Dimer, CA125, anti-β2 glycoprotein-I (aβ2 GPI), and anti-cardiolipin (aCL) IgG/A/M antibodies. The odds ratios (ORs) and 95% confidence intervals (95% CIs) for the logistic regression models were calculated using the “nnet” package in the R software. Sequential mediation analysis was conducted using the “mediate” function from the “Mediation” package. To ensure robustness and reliability, mediation analysis was performed with a bootstrap method involving 100 rounds (Yan et al. 2022).

All statistical analyses were performed using R statistical software version 4.0.3 (R Foundation). All *P* values were two-sided and *P* < 0.05 was considered statistically significant.

## Results

### Basic characteristics of study participants

Our analysis encompassed a total of 311 infertile women undergoing ART treatment. Among the participants, 118 exhibited the normal (CC) genotype of *MTHFR*, 176 had the heterozygous (CT) genotype, and 17 presented the homozygous (TT) genotype (Table 1). Infertile women carrying the *MTHFR* 677T allele (both heterozygous CT and homozygous TT genotypes) demonstrated higher average age, urea levels, and creatinine levels, accompanied by lower AMH levels.

**Table 1:** Clinical characteristics

Characteristics	*MTHFR* 677 allele in infertile women (*n* = 311)			*P*
	CC (*n* = 118)	CT(*n* = 176)	TT (*n* = 17)	
Age	31.6 (4.3)	32.5 (5.0)	32.9 (5.2)	0.01
BMI				0.82
Lean (≤ 18.5)	17 (14.4%)	17 (9.7%)	2 (11.8%)	
Normal weight (18.5–24)	81 (68.6%)	136 (77.3%)	12 (70.6%)	
Overweight/obesity (> 24)	16 (13.6%)	20 (11.4%)	2 (11.8%)	
Urea	3.9 (1.2)	4.1 (1.0)	4.2 (1.0)	0.04
Creatinine	54.1 (8.4)	55.1 (8.4)	57.2 (10.4)	0.02
D-Dimer	0.47 (0.61)	0.60 (2.43)	0.44 (0.52)	0.63
CA125	33.5 (51.8)	27.7 (45.1)	17.5 (8.9)	0.08
aβ2 GPI	5.5 (19.9)	6.01 (22.2)	3.2 (2.03)	0.69
AMH	3.4 (2.4)	2.7 (1.6)	1.8 (1.6)	0.01
aCL IgG	2.7 (1.3)	2.8 (3.4)	3.4 (3.3)	0.97
aCLIgA	2.9 (2.4)	2.6 (0.6)	4.4 (6.0)	0.16
aCLIgM	2.8 (2.3)	2.9 (1.8)	2.7 (1.1)	0.14

*BMI* body mass index, *aβ2 GPI* anti-β2 glycoprotein-I antibody, *aCL* anti-cardiolipin antibody, *CA125* carbohydrate antigen 125, *AMH* anti-Müllerian hormone

Other clinical characteristics, including BMI, D-Dimer, aβ2 GPI, and aCL IgG/A/M antibodies, were comparable across all *MTHFR* genotypes (Table 1).

### The MTHFR 677 T allele and ART efficacy

**Fig. 2:**
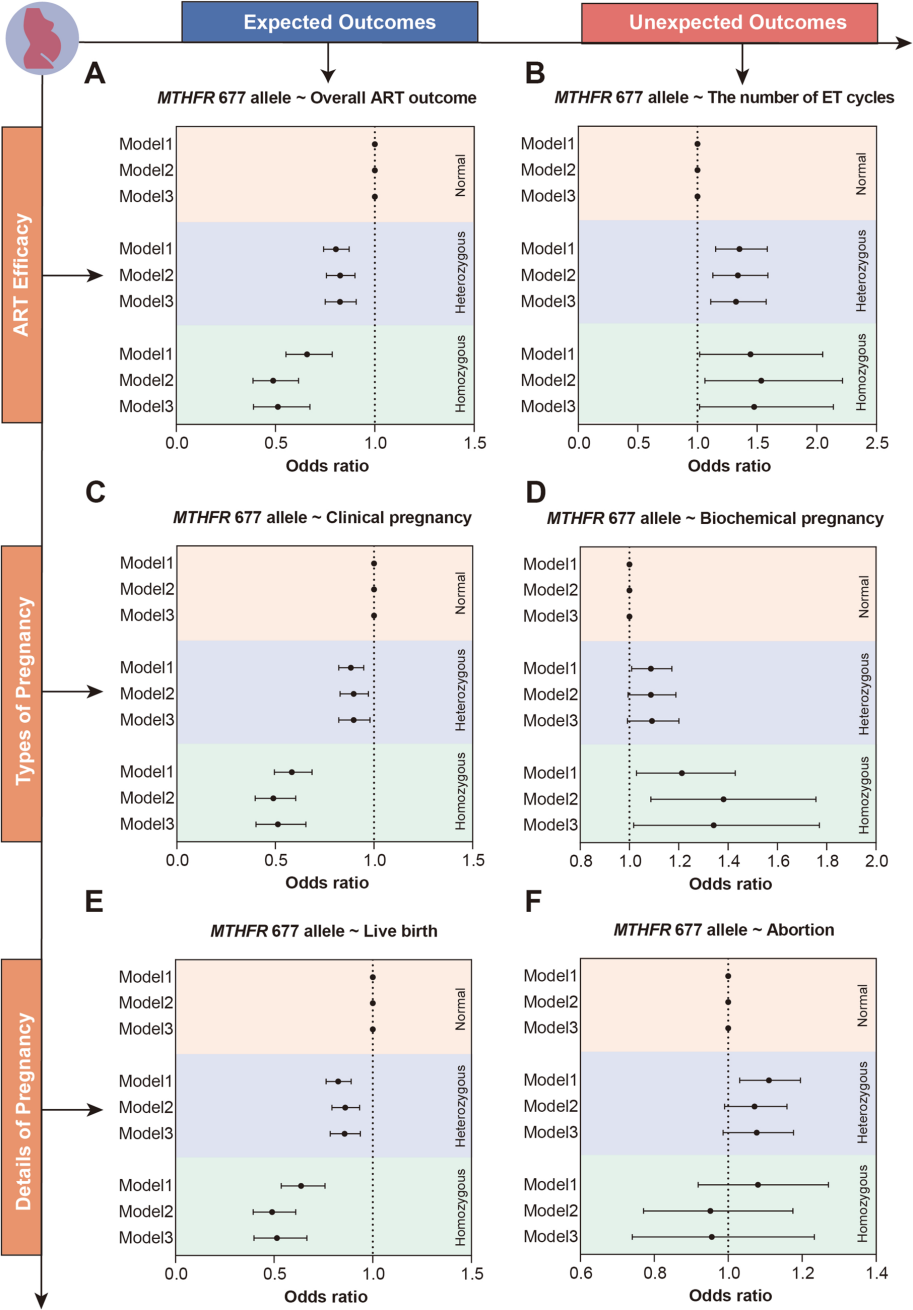
Association between *MTHFR* 677T allele and ART efficacy. The association between *MTHFR* 677T allele and the overall ART outcome (**A**), ART cycles (**B**), clinical pregnancy (**C**), biochemical pregnancy (**D**), live birth (**E**), and abortion (**F**). Model 1 was set as crude model without adjustment; Model 2 was adjusted for age and BMI; Model 3 was adjusted for covariates in Model 2 plus routine laboratory tests including urea, creatinine, D-Dimer, CA125, anti-β2 glycoprotein-I (aβ2 GPI) and anti-cardiolipin (aCL) IgG/A/M antibodies

Considering the comparable prevalence of *MTHFR* 677T allele in both normal pregnancy and infertile females, an important question arises regarding the potential impact of these genetic variations on the effectiveness of ART. By applying a logistic model, we established a link between *MTHFR* 677T allele and a reduced efficacy in ART. Specifically, *MTHFR* 677T allele was significantly associated with a decreased likelihood of achieving favorable outcomes in ART (For heterozygous mutated 677T allele of *MTHFR*: Model 1: crude OR 0.80, 95% CI 0.74–0.87; Model 2: adjusted OR 0.83, 95% CI 0.76–0.90; Model 3: adjusted OR 0.83, 95% CI 0.75–0.91; For homozygous mutated 677T allele of *MTHFR*: Model 1: crude OR 0.66, 95% CI 0.55–0.79; Model 2: adjusted OR 0.49, 95% CI 0.39–0.62; Model 3: adjusted OR 0.51, 95% CI 0.39–0.67) (Fig. 2A). Additionally, *MTHFR* 677T allele was also significantly associated with a higher number of embryo transfer cycles, indicating diminished ART efficacy in both crude and adjusted models (for heterozygous mutated 677T allele of *MTHFR*: adjusted OR 1.33, 95% CI 1.11–1.58; for homozygous mutated 677T allele of *MTHFR*: adjusted OR 1.49, 95% CI 1.02–2.15) (Fig. 2B).

Consistently, we further observed that different *MTHFR* 677T allele were associated with lower rates of clinical pregnancy (Fig. 2C) but higher rates of biochemical pregnancy (Fig. 2D). Moreover, the likelihood of experiencing a miscarriage significantly increased among infertile women with the *MTHFR* 677T allele (Fig. 2E), while their chances of achieving a live birth remained comparatively lower than those with the normal allele of *MTHFR* (Fig. 2F). In summary, we emphasize that the normal allele of *MTHFR* was linked to improved ART outcomes as well as a reduced number of ART treatment cycles.

### The sequential mediation analysis on ART efficacy

**Fig. 3:**
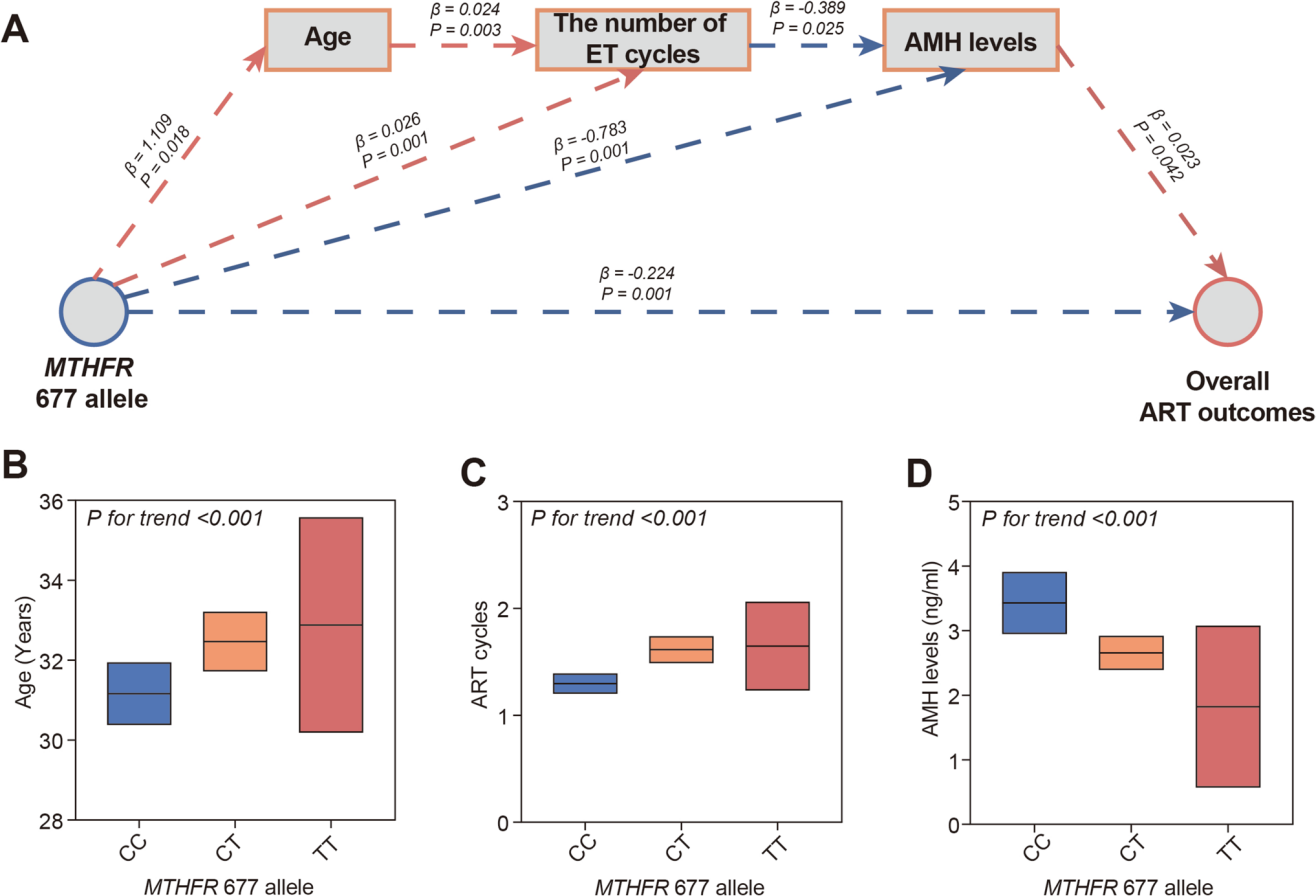
Sequential mediation analysis of *MTHFR* 677T allele and ART efficacy (**A**). The mediating effects of AMH and age in the association between *MTHFR* 677T allele and ART efficacy. The levels of age (**B**), ART cycles (**C**), and AMH levels (**D**) in infertile women with different *MTHFR* genotype

Using an innovative sequential mediation analysis, we explored a broad spectrum of clinical characteristics to identify potential mediators linking the *MTHFR* 677T allele to ART efficacy (Fig. 3A). Our comprehensive analysis highlighted age, the number of embryo transfer (ET) cycles, and AMH levels as significant mediators in this relationship. Specifically, the *MTHFR* 677T allele appears to influence the age at conception, leading to an increased number of ET cycles and lower AMH levels, ultimately resulting in diminished ART outcomes (Fig. 3A). To substantiate these findings, we further evaluated age, ET cycles, and AMH levels in individuals carrying the *MTHFR* 677T allele. Consistent with our previous results, a pronounced graded association was observed between these mediating factors and the *MTHFR* 677T allele (*P* for trend < 0.001 for all) (Fig. 3B-D). Collectively, these findings illuminate the intricate interplay between the *MTHFR* 677T allele and ART efficacy, underscoring the pivotal mediating roles of age and AMH levels.

## Discussion

In a cohort of 311 infertile women undergoing ART treatment, this study is the first to elucidate the relationship between the *MTHFR* 677T allele and ART efficacy, highlighting the pivotal mediating roles of AMH and age in these associations.

Despite substantial clinical and experimental evidence implicating the *MTHFR* 677T allele in adverse pregnancy outcomes, few studies have specifically examined its impact on ART outcomes. Notably, the existing literature has largely overlooked a crucial factor in ART success: the number of treatment cycles (Lu et al. 2023; Haggarty et al. 2006; Morales et al. 2021). Addressing this gap, our study provides the first comprehensive assessment of the association between the *MTHFR* 677T allele and ART efficacy, with a particular focus on treatment cycle frequency. Through meticulous adjustment for a wide range of confounding variables, we identified a strong and consistent link between the *MTHFR* 677T allele and reduced ART success, reflected in poorer overall outcomes and a significant increase in the number of ET cycles required. These findings are consistent with previous research across diverse populations (D’Elia et al. 2014). Furthermore, we found that the *MTHFR* 677T allele is associated with lower clinical pregnancy rates, reduced live birth rates, higher incidences of biochemical pregnancy, and an increased risk of miscarriage. These associations underscore the clinical significance of our findings, highlighting the detrimental impact of the *MTHFR* 677T allele on ART outcomes. The implications of our study extend beyond the immediate effects of this genetic variant on ART, offering broader insights into its role in reproductive health. Given the association of the *MTHFR* 677T allele with increased treatment cycles and reduced clinical success, our findings hold important implications for optimizing the management strategies for patients undergoing ART. The necessity for additional embryo transfer cycles imposes significant emotional and financial burdens on patients and may increase cumulative exposure to ART-related risks, such as ovarian hyperstimulation syndrome or multiple pregnancies. Our findings also prompt consideration of the potential utility of genetic screening for the *MTHFR* 677T allele in pre-treatment assessments for individuals seeking ART. Identifying patients with this allele could enable clinicians to implement personalized treatment strategies, such as targeted folate supplementation or enhanced monitoring during ART cycles, to mitigate its adverse effects. Moreover, the established link between this allele and homocysteine metabolism suggests additional therapeutic opportunities, where addressing elevated homocysteine levels may enhance ART outcomes.

Most studies investigating the relationship between the *MTHFR* 677T allele and ART outcomes have been constrained by a limited exploration of the underlying biological mechanisms, hindering progress in experimental research. In our investigation, we identified AMH as a negative mediator in the association between the *MTHFR* 677T allele and ART efficacy, providing new insights into the impact of the *MTHFR* 677T allele on reproductive outcomes. Our findings reveal a progressive decline in AMH levels among infertile women carrying the *MTHFR* 677T allele, with a significant trend observed in individuals with both CT and TT genotypes. Notably, women with the TT genotype exhibited the lowest AMH levels, indicating a more pronounced impact on ovarian reserve. Elevated AMH levels are known to enhance ovarian responsiveness to stimulation during ART, thereby prolonging reproductive potential and improving live birth rates (Grynnerup et al. 2012; Yang et al. 2016). Thus, the observed reduction in AMH levels among carriers of the *MTHFR* 677T allele (both CT and TT genotypes) may, in part, explain the diminished ART success observed in this population. Moreover, we considered age as an additional mediator, observing that advancing maternal age further diminished the potential protective role of *MTHFR*. This age-related attenuation may be driven by both declining AMH levels and the progressive deterioration of oocyte quality, which are well-documented factors influencing ART outcomes in older women (Pierce and Mocanu 2018). Together, these findings point to a complex interplay between *MTHFR* genotype, AMH levels, and age in shaping ART success, underscoring the importance of addressing these mediators in clinical practice. While our study offers a robust framework for understanding these associations, further experimental validation is crucial to fully elucidate the underlying mechanistic pathways and to confirm the potential of these mediators as therapeutic targets for optimizing ART outcomes in women with the *MTHFR* 677T allele.

It is important to acknowledge the limitations of this study. First, the sample size and the specific population examined may not be fully representative of all infertile women, particularly those without the *MTHFR* 677T allele (Ren et al. 2017; Laanpere et al. 2011). Further research involving larger sample sizes and diverse populations is necessary to validate these findings and provide more robust evidence to guide clinical decision-making. Besides, the specific follow-time of the individuals included in this study was missing, which may contribute to the bias of the constructed model.

## Conclusions

The results emphasize the necessity for personalized treatment strategies for infertile women, that incorporate testing for *MTHFR* 677T allele, assessment of AMH levels, and consideration of the patient’s age. To further advance our understanding and improve the precision and success rates of infertility treatments, future research should encompass larger-scale studies and comprehensive assessments of various genetic and environmental factors.

## Data Availability

The datasets used and/or analyzed during the current study are available from the corresponding author on reasonable request.
